# Short-term exposure to extreme temperature and outpatient visits for respiratory diseases among children in the northern city of China: a time-series study

**DOI:** 10.1186/s12889-024-17814-5

**Published:** 2024-02-01

**Authors:** Ya Wu, Xiaobo Liu, Lijie Gao, Xiaohong Sun, Qianqi Hong, Qian Wang, Zhen Kang, Chao Yang, Sui Zhu

**Affiliations:** 1 Department of Epidemiology and Statistics, School of Medicine, Jinan University, Guangzhou, 510632 China; 2 Shenzhen Center for Disease Control and Prevention, Shenzhen, 518055 China; 3 Department of Environment, Harbin Center for Disease Control and Prevention, Harbin, 150056 China; 4 Department of Physicochemical Laboratory, Harbin Center for Disease Control and Prevention, Harbin, 150056 China; 5 Harbin Center for Disease Control and Prevention, Harbin, 150056 China

**Keywords:** Extreme temperature, Respiratory diseases, Outpatient visits, Distributed lag nonlinear model, Children

## Abstract

**Background:**

Although studies have indicated that extreme temperature is strongly associated with respiratory diseases, there is a dearth of studies focused on children, especially in China. We aimed to explore the association between extreme temperature and children’s outpatient visits for respiratory diseases and seasonal modification effects in Harbin, China.

**Methods:**

A distributed lag nonlinear model (DLNM) was used to explore the effect of extreme temperature on daily outpatient visits for respiratory diseases among children, as well as lag effects and seasonal modification effects.

**Results:**

Extremely low temperatures were defined as the 1st percentile and 2.5th percentile of temperature. Extremely high temperatures were defined as the 97.5th percentile and 99th percentile of temperature. At extremely high temperatures, both 26 °C (97.5th) and 27 °C (99th) showed adverse effects at lag 0–6 days, with relative risks (RRs) of 1.34 [95% confidence interval (CI): 1.21–1.48] and 1.38 (95% CI: 1.24–1.53), respectively. However, at extremely low temperatures, both − 26 °C (1st) and − 23 °C (2.5th) showed protective effects on children’s outpatient visits for respiratory diseases at lag 0–10 days, with RRs of 0.86 (95% CI: 0.76–0.97) and 0.85 (95% CI: 0.75–0.95), respectively. We also found seasonal modification effects, with the association being stronger in the warm season than in the cold season at extremely high temperatures.

**Conclusions:**

Our study indicated that extremely hot temperatures increase the risk of children’s outpatient visits for respiratory diseases. Efforts to reduce the exposure of children to extremely high temperatures could potentially alleviate the burden of pediatric respiratory diseases, especially during the warm season.

**Supplementary Information:**

The online version contains supplementary material available at 10.1186/s12889-024-17814-5.

## Background

Climate change is one of the serious challenges of modern society. The *Lancet* Countdown on health and climate change [1] states that the world’s average temperature has risen by 1.2 °C relative to last century’s levels, and this trend will continue. The number of extreme weather events is increasing [2]; in particular, extremes of hot and cold are becoming more frequent globally [3]. In China, extremely high temperature events have increased significantly since the mid-1990 [4]. From 1991 to 2020, the average value of China’s climate risk index (6.8) increased by 58% compared to 4.3 in 1961–1990 [5]. The correlation between extreme temperatures and a variety of adverse health outcomes, such as morbidity and mortality, is well established [6–8], especially in regards to cardiovascular and respiratory diseases [9–11].

Studies indicated that the atmospheric environment including external meteorological changes, had a direct impact on respiratory diseases, especially in regard to extreme temperatures [8, 12]. There are substantial differences among different populations’ vulnerability to temperature stress [13]. Compared to adults and the elderly, children appear to be more susceptible to temperature changes [14], and might be greater vulnerability due to the ongoing development of their respiratory and immune systems [15]. Although studies [16–20] have reported that extreme temperatures are associated with respiratory diseases among children in different climate regions, the findings are inconsistent. For example, Wen et al. [20] found an inverted U-shape relationship between temperature and respiratory diseases in children in Baotou, China, which indicated that extreme temperatures had no effects on children’s respiratory diseases as evidenced by the relative risks (RRs) of 1.04 (0.93–1.17) and 0.96 (0.85–1.07) at extremely low and high temperatures, respectively. In contrast, another study revealed that both low and high temperatures were associated with an increased risk of respiratory diseases among children, with RRs of 1.082 (95% CI: 1.025–1.142) and 1.099 (95% CI: 1.049–1.152) at extremely low and high temperatures, respectively [16]. Therefore, more similar researches are required to complement the existing evidence from different regions.

Furthermore, studies on the health effects of extreme temperatures have mainly concentrated on mortality or hospital admissions. Conversely, research on the relationship between outpatient visits and extreme temperatures, particularly among children, are relatively scarce [16–20]. Therefore, it is necessary to investigate the influence of extreme temperatures on childhood outpatient visits for respiratory disease, which could provide a foundation for subsequent extensive studies in the field.

Research indicated that individuals from different regions exhibit inconsistent responses to adverse events caused by extreme temperatures [21, 22]. People residing in the southern provinces of China were more susceptible to extreme cold events, whereas those living in the northern provinces tended to be more sensitive to heat waves [22]. Meanwhile, developing cities are more vulnerable to extreme climates than developed cities [23]. In China, there has been few studies on the association between extreme temperatures and respiratory diseases among children, with most studies focused on developed cities and southern regions [24–29]. As a result, there is a need for further research to explore the associations between extreme temperatures and respiratory diseases among children, especially in developing cities located in the northern region of China.

Harbin, which is located in northern China and serves as the capital of Heilongjiang Province, is considered a developing city with a per capita gross domestic product (PGDP) of less than 50,000 yuan in 2022 (http://www.stats.gov.cn/tjsj/ndsj/2022/indexch/). Recently, a new study including three cities in northern China (Shenyang, Changchun, and Harbin), suggested a correlation between extreme temperatures and respiratory diseases [30]. However, the study only included general population from Harbin, and the confounding effects of air pollutants were not considered [30]. Therefore, it is necessary to conduct research in Harbin to fill the aforementioned research gaps.

Understanding the regional association between temperature and respiratory diseases in childhood is crucial for formulating strategic plans aimed at reducing the burden of respiratory diseases, especially given the increased occurrence of extreme weather events. In our study, we aimed to (1) explore the short-term associations between extreme temperature and children’s outpatient visits for respiratory diseases in Harbin using a retrospective time-series approach and (2) evaluate the seasonal modification effect on the association between extreme temperatures and outpatient visits for respiratory diseases.

## Materials and methods

### Study setting

Harbin is the capital city of Heilongjiang Province, located in northern China within a temperate zone and spanning coordinates of 125.4°E to 130.1°E longitude and 44.0°N to 46.4°N latitude. Harbin has a monsoon climate with an annual average temperature of 5.6 °C and covers a land area of approximately 53,186 km² (Fig. S1). According to the seventh national census launched in 2020, the population of children under 18 years old in Harbin is 1.29 million, accounting for 13.5% of the city’s total population (http://tjj.hlj.gov.cn/tjsj/tjnj/).

### Outpatient data of respiratory diseases among children

All children under 18 years old who sought medical help for pediatric respiratory diseases at Harbin Children’s Hospital during the study period were included [31]. Harbin Children’s Hospital is the largest children’s hospital in the three northeastern provinces of China, and also serves as the only comprehensive children’s hospital in Harbin. In this study, data on pediatric respiratory diseases were obtained from the hospital’s health information system and the number of children were from Harbin Center for Disease Control and Prevention between January 1, 2013 and December 31, 2019. The diagnosis of respiratory diseases from outpatient visits was made by the clinicians based on patient’s previous medical history, clinical symptoms and signs, as well as the results of physical and chemical examinations. All medical records coded as ‘J00–J99’, according to the International Classification of Diseases 10th version (ICD-10), were considered respiratory diseases.

### Meteorological and air pollution data

During the study period, we collected daily mean temperature, daily mean relative humidity, and daily mean air pressure from the China Meteorological Data Sharing Service System (http://data.cma.cn/), which included measurements from 8 weather monitoring stations (Fig. S2). To allow for adjustment of covariates, we obtained the data on daily mean values of multiple air pollutants (including sulfur dioxide (SO_2_), nitrogen dioxide (NO_2_), carbon monoxide (CO), and airborne particulate matter with an aerodynamic diameter < 2.5 μm (PM_2.5_) or 10 μm (PM_10_)) from the China National Environmental Monitoring Centre (https://www.cnemc.cn/). These data were obtained from 12 environmental monitoring stations (Fig. S2). The daily average of meteorological factors, SO_2_, NO_2_, CO, PM_2.5_, and PM_10_ were calculated by taking the 24-hour average monitoring records from all weather and environment monitoring stations into account. For missing air pollutant variables, including PM_10_ (*n* = 27), NO_2_ (*n* = 27), and SO_2_ (*n* = 27), we used the Kalman smoothing method to impute the missing values by the R package “imputeTS”.

### Statistical analysis

In the descriptive analyses, the mean, standard deviation (SD), quartiles (P_25_, P_50_, P_75_), minimum (min), and maximum (max) were calculated to describe the distribution of the daily number of outpatient visits as well as the meteorological variables. We used a distributed lag nonlinear model (DLNM) based on a quasi-Poisson regression to assess the impact of extreme temperatures on outpatient visits for respiratory diseases among children. The natural cubic spline (*ns*) function of time was used to adjust the long-term and seasonal trends with 7 degrees of freedom (*df*) per year [32]. Meanwhile, other potential confounding factors such as the day of the week (DOW) effect and the impact of public holidays, were also controlled. Both public holidays and DOW were classified as categorical variables. To ensure the robustness of our time series analysis, the model’s validity was contingent upon satisfying the white noise assumption. Accordingly, we performed a Box test to examine this aspect. The results of this test indicated that the errors in our model conform to the white noise assumption (*P* > 0.05).

To adjust for the potential effects of other factors, such as air pollutants and other meteorological factors, we adjusted for PM_10_, SO_2_, NO_2_, and humidity, which was consistent with previous studies [33, 34]. We calculated the Spearman rank correlation coefficients between the variables and observed a strong correlation between air pollutants (Fig. S3). To assess collinearity, we calculated the variance inflation factor (VIF) values of the independent variables, which included PM_10_, SO_2_, NO_2_, temperature, and humidity [35]. Based on the VIF values, which were lower than 5, we can conclude that there was no underlying collinearity among the independent variables (Table S1).

Finally, we incorporated several covariates: (1) a *ns* function of time with 7 *df* per year to control for seasonality and long-term trends; (2) a *ns* function with 3 *df* for relative humidity, PM_10_, NO_2_, and SO_2_ at the current day [36], which had the highest goodness of quasi-likelihood model fit with the lowest modification of Akaike Information Criterion (AIC); (3) a categorical variable for DOW; and (4) a binary dummy variable for public holidays. The model was described as follows:

$${Y_t} \sim Poisson({\mu _t})$$

1 $$\eqalign{& Log\left( {{\mu _t}} \right) = \alpha+ \beta \left( {tem{p_{t,l}}} \right) \cr &+ ns\left( {tim{e_t},\,7 * 7} \right) + ns\left( {rh,\,3} \right) \cr &+ ns\left( {P{M_{10}}_,\,3} \right) + ns\left( {N{O_2}_,\,3} \right) \cr &+ ns\left( {S{O_2}_,\,3} \right) + DO{W_t} \cr &+ \,Holida{y_t}\, + \,po{p_t} \cr} $$

In Eq. (1), $$ {Y}_{t} $$ represents the daily number of children’s outpatient visits for respiratory diseases on Day *t*, *α* is the intercept, $$ {temp}_{t,l} $$is a matrix obtained by applying the DLNM to temperature, *β* is a vector of coefficients for $$ {temp}_{t,l}$$, and *l* represents the lag days. $$ {DOW}_{t}\,\text{a}\text{n}\text{d}\,{Holiday}_{t}$$ are categorical variables used to control for day of the week and public holiday effects, respectively. $$ {pop}_{t}$$ is the offset variable, which is the number of children in the *t* year.

We defined the cross-basis matrices for temperature using the B-spline (*bs*) function with internal knots located at the 25th, 50th, and 75th percentiles. Regarding the space of lags, we evaluated time lags ranging from 0 to 27 days according to a previous study [37]. The max lag is determined by a lag-response plot (Fig. S4), which shows that the max lag period can be extended to 6 days and 10 days for the hot effect and cold effect, respectively. In addition, considering that both cold and hot effects were not significant at a lag of 2 days, we also analyzed the effect with a lag of 0–1 days. The knots for lags are placed at equally spaced log values of lag using the function lognots. RRs were calculated using 7 °C (median temperature) as a reference value [33]. We computed the 1st, 2.5th, 97.5th, and 99th percentiles of the temperature (− 23, -26, 26, and 27 ℃), and considered them as the extremely low and high temperatures to explore the effects of cold and hot, respectively [38–40]. The lag effects for lags of 1 and 6 at specific temperatures (26 and 27 °C) were calculated to estimate the cumulative effects of extremely high temperatures. The lag effects for lags of 1 and 10 at specific temperatures (-23 and − 26 °C) were calculated to estimate the cumulative effects of extremely low temperatures.

To analyze the effects of seasonal modification, we divided the data into the warm season (April to September) and cold season (October to March) [41] and the model differed slightly from Eq. (1). Specifically, *ns* with 3 *df* per season (6 months) was used to adjust for long-term trends. We statistically tested the seasonal modification effect on the impact of extreme temperatures on outpatient visits for respiratory diseases. We calculated the 95% confidence interval (CI) for the relative difference in RRs between the warm and cold seasons. The relative difference in RRs refers to the ratio of two RRs, which was regarded as the relative risk ratio (RRR). The formula for calculating RRR is as follows [42]:

2 $$ exp\left[\left({E}_{1}-{E}_{2}\right)\pm 1.96\sqrt{{SE}_{1}^{2}+{SE}_{2}^{2}}\right]$$

We conducted a *Z*-test to test the statistical significance of RRR, as shown below [43]:

3 $$ Z=\frac{{E}_{1}-{E}_{2}}{\sqrt{{SE}_{1}^{2}+{SE}_{2}^{2}}} $$

In the above equation, $$ {E}_{1}$$ and $$ {E}_{2}$$ refer to ln(*RR*) in the cold and warm seasons, respectively, while *SE*_1_ and *SE*_2_ refer to their corresponding standard errors [44].

Sensitivity analyses were applied to verify the robustness of associations between extreme temperatures and outpatient visits for children’s respiratory diseases reported in the primary model. These analyses involved the following adjustments: (1) increasing the *df* from 6 to 10 for time in the models; (2) modifying the *df* of *ns* functions for relative humidity from 1 to 7; and (3) adjusting for the impacts of CO and other air pressure, based on the primary model.

All statistical tests were two-tailed, and *p* values < 0.05 were defined as statistically significant. We conducted all statistical analyses using R Software (Version 3.6.1).

## Results

### Descriptive results

Between January 1, 2013, and December 31, 2019, there were a total of 10,57521 daily outpatient visits to Harbin Children’s Hospital for respiratory diseases. The daily average number of outpatient visits was 414 cases, with a range of 62 to 1,407. The average daily temperature and relative humidity in Harbin during the study period were 4.51 °C and 65.43%, respectively. The mean concentrations of PM_10_, NO_2_, and SO_2_ were 83.17 µg/m^3^, 43.74 µg/m^3^, and 31.47 µg/m^3^, respectively. The average daily number of outpatient visits, mean temperature, relative humidity, and concentrations of air pollutants during the warm and cold seasons are shown in Table 1. The daily distribution of children’s outpatient visits for respiratory diseases, weather conditions, and concentrations of air pollutants during the study period are shown in Fig. S5. The concentrations of air pollutants (PM_10_, NO_2_, and SO_2_) showed seasonal trend, with higher concentrations in winter and lower concentrations in summer. Conversely, the temperature was higher in summer and lower in winter. However, there was no regular trend in the daily number of outpatient visits for children with respiratory diseases during the study period.

**Table 1:** Descriptive statistics of outpatient visits for children with respiratory diseases, weather conditions, and concentrations of air pollutants by seasons in Harbin from January 1, 2013, to December 31, 2019

Season	Variables	Mean	SD	Min	P_25_	P_50_	P_75_	Max
Whole year	Daily counts, mean	413.74	198.94	62.00	244.00	400.50	537.00	1407.00
	Mean temperature (°C)	4.51	15.55	-33.50	-10.30	7.10	18.41	30.80
	Relative humidity (%)	65.43	14.91	15.00	56.50	67.00	76.00	98.00
	PM_10_ (µg/m^3^)	83.17	67.56	1.00	42.00	65.00	101.05	844.92
	N0_2_ (µg/m^3^)	43.74	20.64	10.00	29.00	39.00	54.00	161.17
	SO_2_ (µg/m^3^)	31.47	35.73	2.82	8.50	16.96	41.00	233.36
Warm	Daily counts, mean	391.65	175.77	96.00	248.00	385.00	496.00	1407.00
	Mean temperature (°C)	17.36	6.48	-3.20	13.30	18.40	22.20	30.80
	Relative humidity (%)	66.64	17.29	15.00	56.00	70.00	80.00	98.00
	PM_10_ (µg/m^3^)	63.76	45.12	11.00	35.00	51.70	79.17	518.00
	N0_2_ (µg/m^3^)	34.32	13.11	10.00	25.00	32.00	40.00	107.83
	SO_2_ (µg/m^3^)	11.06	6.79	2.82	7.00	9.00	13.00	48.58
Cold	Daily counts, mean	435.94	217.59	62.00	242.50	414.00	582.00	1217.00
	Mean temperature (°C)	-8.40	10.50	-33.50	-16.60	-10.30	0.30	20.65
	Relative humidity (%)	64.22	11.95	19.00	56.80	65.00	73.00	97.00
	PM_10_ (µg/m^3^)	102.66	79.69	1.00	53.00	79.00	124.52	844.92
	N0_2_ (µg/m^3^)	53.19	22.43	11.00	37.00	50.00	65.23	161.17
	SO_2_ (µg/m^3^)	51.97	40.92	4.91	23.61	40.92	66.50	233.36

### Exposure-lag-response relationship

**Fig. 1:**
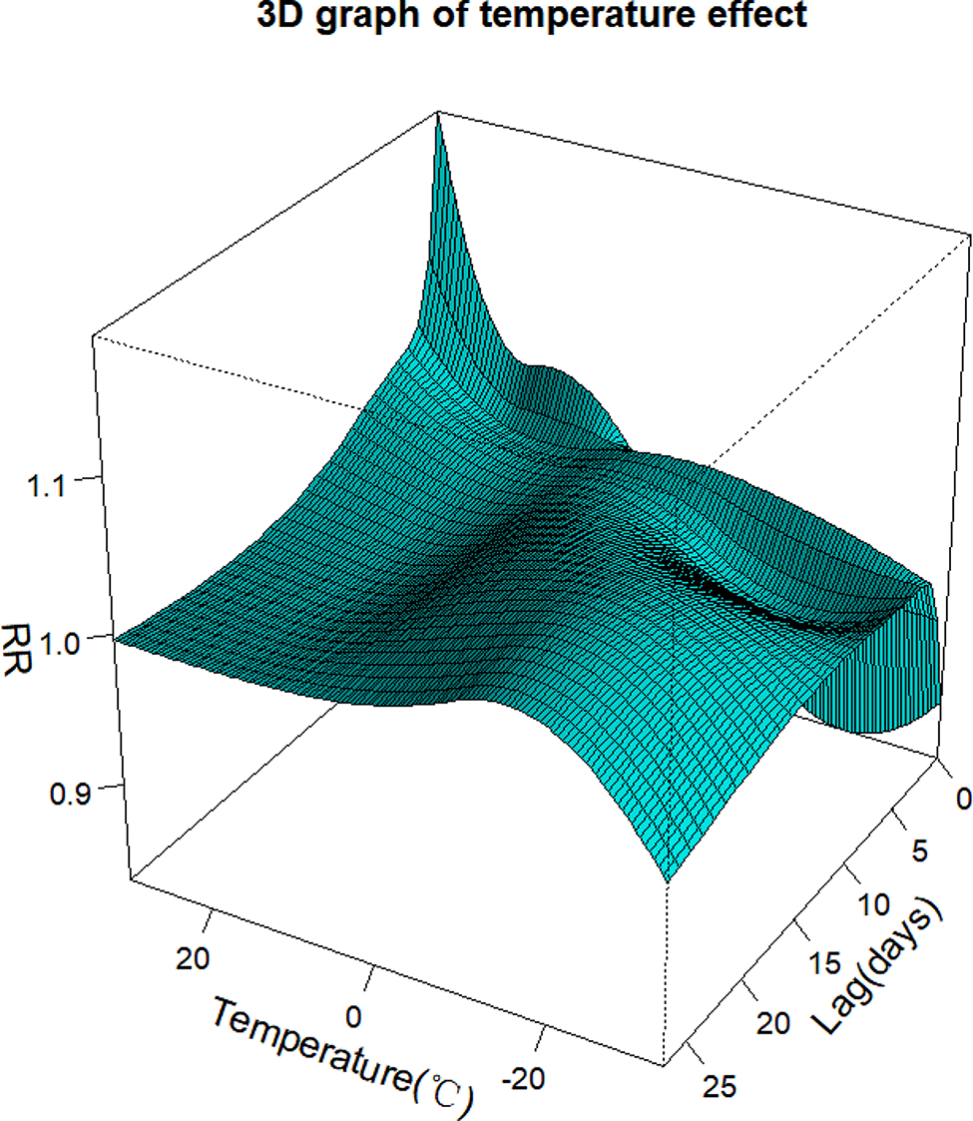
3-D graph of the RR with temperature and lag compared with a reference temperature of 7 °C

Figure 1 displays the 3-D exposure-response plot of the mean temperature on daily outpatient visits for respiratory diseases along lag days. Overall, the effect of temperature on the risk of daily outpatient visits for children’s respiratory diseases was nonlinear. From the 3-D plot, we found that extremely high temperatures presented a harmful effect, while extremely low temperatures presented a protective effect. The harmful effects of the hot effect lasted for approximately one week, and the effect decreased rapidly with the increase in lag days. Compared to the hot effect, the protective effect of the cold effect lasted approximately two weeks, and the effect gradually diminished with the increase in lag days.

**Fig. 2:**
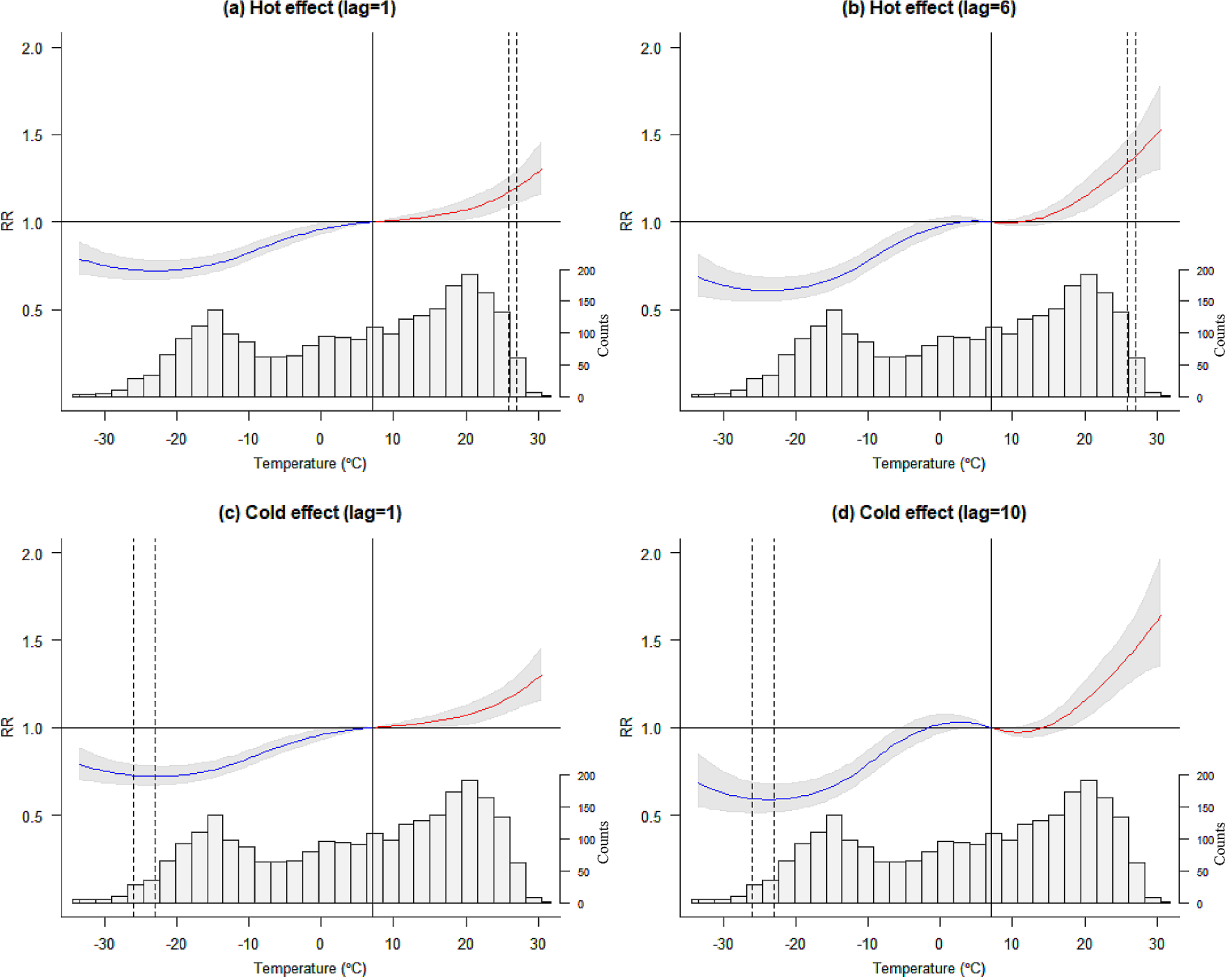
The overall exposure-response relationship between temperature and daily outpatient visits for children’s respiratory diseases at extremely high and low temperatures over different lag days. The vertical dashed lines from left to right represent the 97.5th and 99th percentiles of the temperature, respectively (**a**, **b**); the vertical dashed lines from left to right represent the 1st and 2.5th percentiles of the temperature, respectively (**c**, **d**); the reference level is at 7 °C (50th percentile), indicated by the solid vertical line

The overall exposure-response relationship between temperature and daily outpatient visits for children’s respiratory diseases showed a nonlinear curve with a reference of 7 °C (shown in Fig. 2). The histogram in the graph reflects the distribution of the number of outpatient visits for children’s respiratory diseases at different temperatures. Hot exposure on outpatient visits for children’s respiratory diseases showed harmful effects at both lag 0–1 day and lag 0–6 days. In contrast, cold exposure on outpatient visits for children’s respiratory diseases showed a protective effect at both lag 0–1 days and lag 0–10 days.

The RRs of hot exposure on outpatient visits for children’s respiratory diseases at lag 0–1 day were 1.17 (95% CI: 1.09–1.26) and 1.20 (95% CI: 1.10–1.29) for extremely high temperatures (97.5th and 99th percentiles, respectively). Extremely high temperature was still a risk factor when the lag time was extended to 6 days, with RR values of 1.34 (95% CI 1.21–1.48) and 1.38 (95% CI 1.24–1.53). The RRs of cold exposure on outpatient visits for children’s respiratory diseases at lag 0–1 day were 0.73 (95% CI: 0.67–0.79) and 0.72 (95% CI: 0.67–0.78) for extremely low temperatures (1st and 2.5th percentiles, respectively). In addition, extremely low temperature was still a risk factor when the lag time was extended to 10 days. Detailed results are presented in Table 2.

**Table 2:** The cumulative effects (RR and 95% CI) of extreme temperature on children’s outpatient visits for respiratory diseases, with the 1st, 2.5th, 97.5th, and 99th percentiles of the daily mean temperature relative to the reference temperature of 7 °C at different lag days in Harbin

	1st (-26 °C)	2.5th (-23 °C)	97.5th (26 °C)	99th (27 °C)
Lag 0–1	0.73 (0.67, 0.79)	0.72 (0.67, 0.78)	1.17 (1.09, 1.26)	1.20 (1.10, 1.29)
Lag 0–2	0.71 (0.64, 0.78)	0.70 (0.64, 0.76)	1.19 (1.10, 1.29)	1.22 (1.12, 1.32)
Lag 0–3	0.68 (0.61, 0.75)	0.67 (0.61, 0.74)	1.23 (1.13, 1.34)	1.26 (1.15, 1.38)
Lag 0–4	0.65 (0.59, 0.73)	0.65 (0.59, 0.72)	1.29 (1.18, 1.41)	1.32 (1.20, 1.46)
Lag 0–5	0.63 (0.56, 0.71)	0.63 (0.57, 0.70)	1.32 (1.20, 1.45)	1.35 (1.22, 1.50)
Lag 0–6	0.61 (0.54, 0.69)	0.61 (0.54, 0.68)	1.34 (1.21, 1.48)	1.38 (1.24, 1.53)
Lag 0–7	0.60 (0.52, 0.68)	0.60 (0.53, 0.67)	1.35 (1.22, 1.50)	1.39 (1.24, 1.55)
Lag 0–8	0.59 (0.52, 0.68)	0.59 (0.52, 0.67)	1.36 (1.22, 1.52)	1.40 (1.25, 1.58)
Lag 0–9	0.59 (0.51, 0.68)	0.59 (0.52, 0.67)	1.38 (1.23, 1.54)	1.42 (1.26, 1.61)
Lag 0–10	0.59 (0.51, 0.69)	0.59 (0.51, 0.68)	1.40 (1.25, 1.58)	1.45 (1.28, 1.65)

### Modification effect

Generally, a modification effect by season was found on associations between extremely high temperature and children’s outpatient visits for respiratory diseases (*p* < 0.05), with stronger risk associations in the warm season than in the cold season, especially for extremely high temperatures (Table 3). The critical values (1st, 2.5th, 97.5th, 99th) of temperature during the warm season were 0 °C, 2 °C, 27 °C, and 28 °C, respectively, while during the cold season, they were − 28 °C, -26 °C, 12 °C, and 14 °C, respectively. A statistically significantly modification effect by season on the association between extremely high temperatures and children’s outpatient visits for respiratory diseases (*p* < 0.05), with a stronger effect in the warm season than in the cold season for extremely high temperatures (99th) and a cumulative lag of 0–6 days. The RRs were 1.15 (95% CI: 1.05–1.26) in the warm season and 0.98 (95% CI: 0.87–1.12) in the cold season, with an RRR of 1.12 (1.07, 1.43), indicating a 12% increase in the RR in the warm season compared to the cold season.

**Table 3:** Cumulative effect (RR and 95% CI) of extreme temperature (1st, 2.5th, 97.5th and 99th percentiles) on children’s outpatient visits for respiratory diseases stratified by cold and warm seasons, with respect to the reference of median of daily average temperature at different lag days in Harbin

Temperature percentiles	Lag (day)	Warm season		Cold season		Difference test	*z*	*p*
		RR (95% CI)		RR (95% CI)		RRR (95% CI)		
1st	Lag 0–1	1.02 (0.95, 1.10)		1.00 (0.94, 1.05)		1.08 (0.98, 1.19)	0.58	0.56
	Lag 0–6	1.00 (0.91, 1.10)		0.99 (0.93, 1.06)		1.22 (1.06, 1.40)	0.17	0.86
	Lag 0–10	1.06 (0.95, 1.17)		0.98 (0.92, 1.05)		1.17 (0.94, 1.47)	1.12	0.26
2.5th	Lag 0–1	1.01 (0.92, 1.10)		1.00 (0.94, 1.06)		1.07 (0.99, 1.16)	0.23	0.82
	Lag 0–6	1.00 (0.90, 1.12)		0.99 (0.91, 1.07)		1.17 (1.05, 1.30)	0.23	0.82
	Lag 0–10	1.05 (0.93, 1.19)		0.98 (0.90, 1.07)		1.10 (0.97, 1.26)	0.94	0.35
97.5th	Lag 0–1	1.08 (1.02, 1.13)		1.02 (0.94, 1.12)		1.04 (0.96, 1.13)	1.01	0.31
	Lag 0–6	1.11 (1.04, 1.18)		0.98 (0.88, 1.10)		1.25 (1.10, 1.41)	1.88	0.06
	Lag 0–10	1.09 (1.02, 1.18)		0.98 (0.87, 1.10)		1.45 (1.26, 1.68)	1.61	0.11
99th	Lag 0–1	1.10 (1.03, 1.17)		1.03 (0.93, 1.13)		1.05 (0.96, 1.15)	1.16	0.25
	Lag 0–6	1.15 (1.05, 1.26)		0.98 (0.87, 1.12)		**1.24 (1.07, 1.43)**	**1.99**	**0.04**
	Lag 0–10	1.12 (1.01, 1.25)		0.98 (0.85, 1.12)		1.44 (1.21, 1.72)	1.65	0.10

### Sensitivity analysis

Sensitivity analysis was conducted by changing the *df* for time (6 to 10 per year), and relative humidity (1 to 7), and similar results were obtained to those of the original analysis (Table S2-S3), which showed that extremely high temperatures increased the risk of children’s outpatient visits for respiratory diseases, and extremely low temperatures showed a protective effect. Furthermore, the results remained stable after incorporating other potential covariables (CO, air pressure and PM_2.5_) into the primary model, indicating the relative robustness of the primary model applied in our study (Table S4). Specifically, the RRs for hot exposure on outpatient visits for children’s respiratory diseases were 1.33 (95% CI: 1.20–1.47) and 1.37 (95% CI: 1.23–1.52) for extremely high temperatures (97.5th and 99th percentiles, respectively), after adjusting for CO, air pressure and PM_2.5_. For extremely low temperatures (1st and 2.5th percentiles), the RRs for cold exposure on outpatient visits for children’s respiratory diseases were 0.67 (95% CI: 0.57–0.78) and 0.67 (95% CI: 0.58–0.77), respectively.

## Discussion

We explored the short-term effects of extreme temperatures on children’s outpatient visits for respiratory diseases in Harbin, China, during the years 2013–2019. The relationship between temperature and children’s outpatient visits for respiratory diseases was found to be nonlinear in our study. Extremely low temperatures (-26 °C and − 23 °C) were found to have protective effects on daily outpatient visits for children with respiratory diseases at lag 0–1 day and lag 0–10 days, with reductions of 27-41%. However, at extremely high temperatures, both 26 °C (97.5th) and 27 °C (99th) showed adverse effects at lag 0–1 day and lag 0–6 days, with 17%, 20%, 34%, and 38% increases in children’s outpatient visits for respiratory diseases, respectively. A modification effect by season was found for associations of extreme temperatures with children’s outpatient visits for respiratory diseases. Specifically, the hot effect was particularly greater during the warm season than during the cold season.

Our research found a nonlinear relationship between temperature and respiratory diseases, and it showed an S-shaped curve relationship. Many studies have described nonlinear associations between temperature and respiratory disease [18, 34, 45], which was consistent with our study. Notably, the majority of studies have reported associations that take the form of V-shaped, J-shaped, or U-shaped curves [18, 34, 45]. The exposure-response relationships were significantly influenced by various factors, such as the climatic region [46, 47], geographic location [48], and living conditions [49]. China is a vast country, and the climatic characteristics of different regions vary significantly. Specifically, the topography of Lanzhou, as described in a study [18], falls under the category of valley basin type, whereas the region we studied features as a plain topography. Furthermore, the cities of Chengdu [34] and Harbin, located in the southern and northern regions of China, respectively, exhibit distinct climatic characteristics. Because of the diversity of geographic, socioeconomic, and climatic factors, the effects of temperature on respiratory diseases show significant variation across distinct study regions [41].

Our study indicated that extremely hot temperatures increased the risk of children’s outpatient visits for respiratory diseases. This finding consistent with previous studies [50, 51], which has indicated that under the background of global warming, the harmful effects of heat on human health are becoming increasingly serious. Elevated temperatures raise the surface temperature of the skin, reduce the heat dissipation function, and lead to an increase in the internal temperature of the human body, consequently rendering the organs and systems unable to operate normally [52]. In addition, extremely high temperatures have been known to cause imbalances in body temperature regulation, which are often characterized by heightened heart and respiratory rates, as well as damage to vital organs such as the heart, lungs, kidneys, and liver [53]. In the case of children, their bodies are more vulnerable to external environmental factors due to weaker immunity. When extremely high temperatures occur, they tend to more susceptible to respiratory diseases [48], owing to the rapid effect of external temperature on their bodies. Moreover, our analysis revealed that the impact of high temperatures, referred to as the ‘hot effect’, predominantly persisted for approximately one week, with its intensity diminishing rapidly as the lag period increased. This pattern aligns with existing research indicating that high temperatures typically elicit acute physiological responses [50]. As time progresses, the detrimental impact of these hot effects tends to decrease. Consequently, this observation underscores the importance of focusing on the acute implications of high temperatures. Prompt detection and appropriate treatment during these critical initial days are essential to mitigate the adverse health effects associated with extreme heat.

Our research showed a protective effect on respiratory diseases at extremely cold temperatures which was contrary to the findings of previous studies [16, 36, 54]. One possible explanation for the inconsistent results could be the variation in extremely low temperatures between our study area and other study locations. For example, the extremely low temperatures reported (1st) in Lanzhou [16] and Guangzhou [54] were − 16 °C and 8.2 °C, respectively, which are higher than the extremely low temperature observed in our study (-26 °C). It is possible that extreme cold temperature events lower the number of outpatient visits, due to the convenience and accessibility of hospitalization in colder areas [55], which could contribute to the inconsistent findings. Although cold temperatures may trigger childhood respiratory disease (e.g., asthma), due to the cooling of airways on cold days, which may aggravate inflammation, leading to airway narrowing [56], the impact of temperature will be affected by regional customs and habits [57]. The inconsistent results may be due to the distinctive climatic characteristics and living habits in different regions [19]. The geographical location of Harbin determines that it is characterized by lengthy winters and short summers. In Harbin, the average temperature in January fluctuates between − 15 °C and − 30 °C, and the lowest temperature ever recorded was − 37.7 °C. To avoid severe cold weather, residents in Harbin have central heating equipment installed in their homes, and typically stay indoors and use heating equipment from October onwards when the weather is cold [58, 59]. Reduced outdoor activity during periods of extremely low temperatures has resulted in decreased frequency of exposure. In addition, people residing in cold regions have displayed an increased capacity to adapt to cold exposure [14]. Therefore, these factors could potentially explain why the extremely low temperatures in Harbin is a protective factor for respiratory diseases.

Additionally, it is noteworthy that, in comparison to the transient nature of the hot effect, the protective effect associated with cold temperatures was observed to last for approximately two weeks. This effect also showed a gradual decrease in intensity over time as the lag period increased. A plausible explanation for this extended protective effect could be attributed to the predominantly cold climate of Harbin. The residents in this region have developed a heightened awareness and adaptation to cold conditions, which might contribute to this prolonged protective effect against respiratory diseases. However, further investigation is warranted in the future to verify the underlying mechanisms.

Our results suggested that the effects of extremely high temperatures on children’s outpatient visits for respiratory disease were more significant during the warm season than during the cold season. To the best of our knowledge, this is the first study to explore the modifying effect of seasons on the association between extreme temperatures and respiratory diseases among children, which has rarely been examined in previous studies [24, 26]. However, the underlying mechanisms with respect to seasonal modification on the association between extreme temperatures and respiratory disease remain unclear, and the quantity of relevant studies is limited. Therefore, additional epidemiological studies should be conducted to explore the underlying mechanisms of seasonal-specific effects on the associations between extreme temperatures and respiratory disease.

Our study has several limitations. Firstly, the ecological design limits the transferability of the results to the individual level, and it may introduce the potential ecological fallacy. Secondly, the impact of extreme temperatures on acute respiratory disease may be more pronounced compared to chronic respiratory disease. However, our study is limited to the daily count of children’s outpatient visits for respiratory diseases without specific disease information. Thus, we cannot distinguish whether the disease is chronic or acute, which could potentially affect the accuracy and specificity of the results. Future studies should aim to address the limitation of not being able to differentiate between chronic and acute respiratory diseases. By conducting disease-specific analyses, researchers can examine the differential impacts of extreme temperatures on acute and chronic respiratory diseases separately. Thirdly, due to the limited availability of data, we exclusively selected one hospital as the primary study site, which may lead to selection bias and limit the generalization of the findings to a broader population. Last but not least, this study was only conducted in a single city, and the results might not be generalized to other areas due to dissimilarities in population behaviors, geographical location, and weather conditions.

What’s more, it is a challenge to address the issue of underreporting of respiratory disease, especially in children who do not regularly attend medical check-ups at medical institutions. To address this and enhance the scope of future research, we propose the following additional strategies: (1) Implementation of educational and awareness campaigns: We recommend that relevant departments initiate campaigns to elevate awareness among parents and guardians about pediatric respiratory diseases. Emphasizing the importance of timely medical treatment in these campaigns could lead to more accurate disease case reporting. (2) Collaboration with community and educational entities: Establishing partnerships with community health service centers, schools, and kindergartens for effective information sharing is crucial. Such collaborations could yield more comprehensive data on respiratory diseases in children, thereby reducing underreporting. (3) Development of a comprehensive monitoring system: There is a need to establish a detailed monitoring system for pediatric respiratory diseases, encompassing data collection and analysis mechanisms. This would not only improve the tracking and monitoring of disease occurrence but also facilitate the timely detection and resolution of underreporting issues. Incorporating these suggestions into future research could significantly enhance our understanding of respiratory diseases, particularly in the context of their relationship with extreme temperature variations.

## Conclusions

The results of our study indicate that extremely high temperatures increase the risk of outpatient visits for respiratory diseases among children, while extremely low temperatures show a protective effect in northern China. This study provides evidence of the short-term effects of extreme temperature on outpatient visits for respiratory disease and can provide recommendations for public health intervention. Efforts to reduce children’s exposure to extremely high temperatures could potentially alleviated the burden of pediatric respiratory diseases, especially during the warm season. However, large-scale, multicenter, and multinational prospective investigations are warranted to confirm the findings of our study.

### Electronic supplementary material

Below is the link to the electronic supplementary material.

**Supplementary Material 1:** Supplementary tables and figures

## Data Availability

The datasets used and/or analysed during the current study are available from the corresponding author on reasonable request.

## Declarations

### Statement

We confirm that all methods were carried out in accordance with relevant guidelines and regulations.
