# Supplementary material for: Short-term exposure to extreme temperature and outpatient visits for respiratory diseases among children in the northern city of China: a time-series study
Source: BMC Public Health. 2024 Feb 1;24:341. doi: 10.1186/s12889-024-17814-5 (PMC10832290; doi:10.1186/s12889-024-17814-5)
Supplement: Supplementary file 1 — Supplementary Material 1: Supplementary tables and figures [file 12889_2024_17814_MOESM1_ESM.docx]

***Supplementary tables and figures***

**Short-term exposure to extreme temperature and outpatient visits for respiratory diseases among children in the northern city of China: A time-series study**

Ya Wu ^1,2^ **^†^**, Xiaobo Liu ^3,^ **^†^**, Lijie Gao ^1^, Xiaohong Sun ^4^, Qianqi Hong ^3^, Qian Wang ^1^, Zhen Kang ^3^, Chao Yang ^5, *^, Sui Zhu ^1, *^

*** Corresponding authors:**

Harbin Center for Disease Control and Prevention, Harbin 150056, China;

E-mail address: yangchao0608@126.com (Chao Yang);

Department of Epidemiology and Statistics, School of Medical, Jinan University, Guangzhou 510632, China;

E-mail address: zhusui1213@jnu.edu.cn (Sui Zhu).

**Table of Contents**

**Fig.S1.** Geospatial location map of Harbin, China

**Fig.S2.** Spatial distribution of air quality and meteorological monitoring stations in Harbin, Heilongjiang province (2013-2019)

**Fig.S3.** Spearman rank correlation coefficients among air pollutants, meteorological data, and outpatient visits for respiratory diseases among children in Harbin, 2013-2019

**Fig.S4.** (a) The RRs and 95% CIs of 97.5^th^ percentile for temperatures relative to median temperature (7 ℃) as a reference value on children’s outpatient visits for respiratory diseases at different lag days. (b) The RRs and 95% CIs of 99^th^ percentile for temperatures relative to median temperature (7 ℃) as a reference value on children’s outpatient visits for respiratory diseases at different lag days. (c) The RRs and 95% CIs of 2.5^th^ percentile for temperatures relative to median temperature (7 ℃) as a reference value on children’s outpatient visits for respiratory diseases at different lag days. (d) The RRs and 95% CIs of 1^st^ percentile for temperatures relative to median temperature (7 ℃) as a reference value on children’s outpatient visits for respiratory diseases at different lag days.

**Fig.S5.** Daily distribution of air pollutants, meteorological factors, and outpatient visits for respiratory diseases among children in Harbin, 2013-2019

**Table S1.** Variance inflation factor (VIF) values of independent variables included in the model in Harbin2013-2019

**Table S2.** The cumulative effect (lag 0-10 days) of extreme temperatures relative to median temperature (7 ℃) as a reference value on children’s outpatient visits for respiratory diseases using different *df* for calendar time

**Table S3.** The cumulative effect (lag 0-10 days) of extreme temperatures relative to median temperature (7 ℃) as a reference value on children’s outpatient visits for respiratory diseases using different *df* for relative humidity

**Table S4.** The cumulative effect (lag 0-10 days) of extreme temperatures relative to median temperature (7 ℃) as a reference value on children’s outpatient visits for respiratory diseases using the covariate-adjusted standardization approach

**
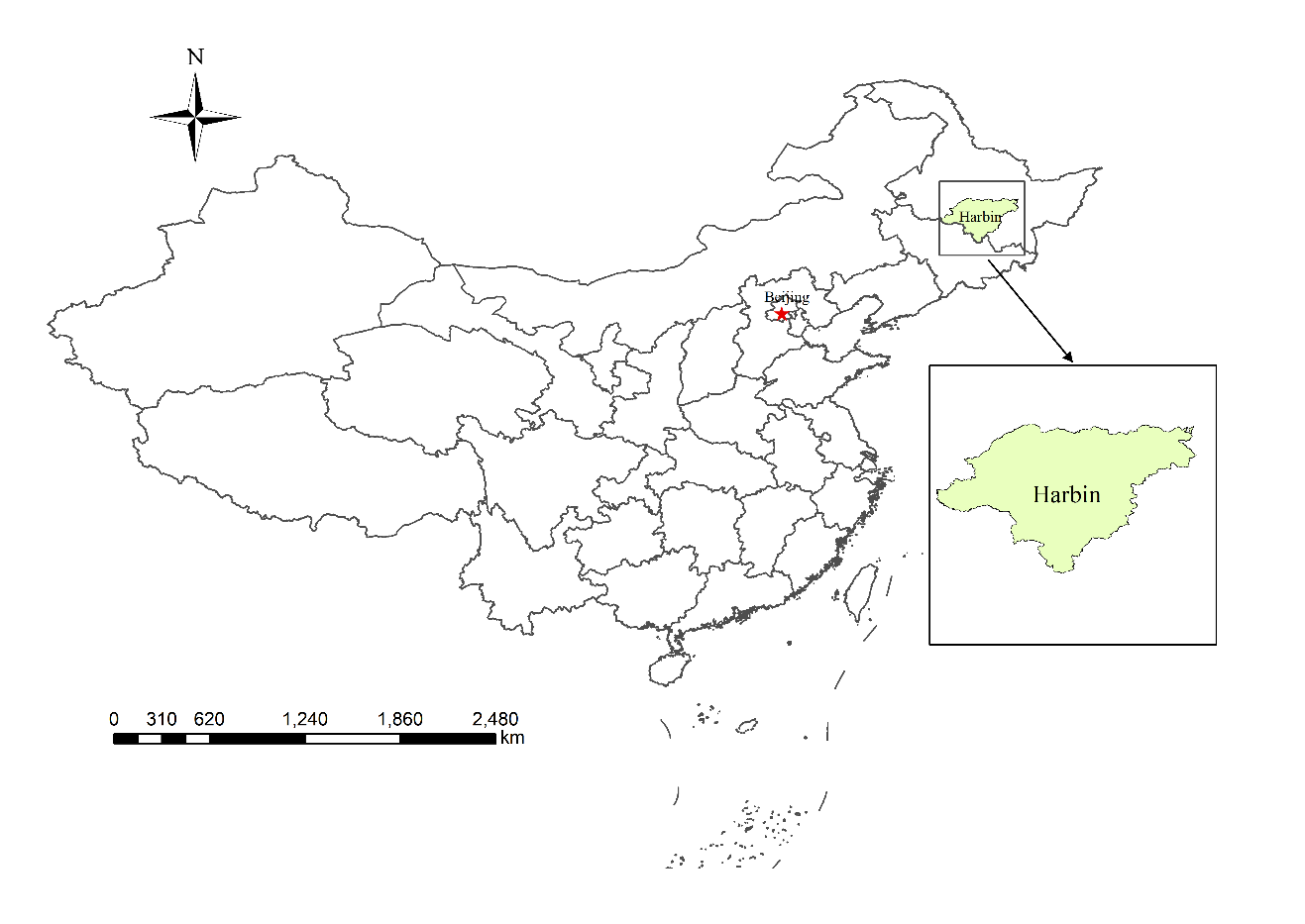
**

**Fig.S1.** Geospatial location map of Harbin, China


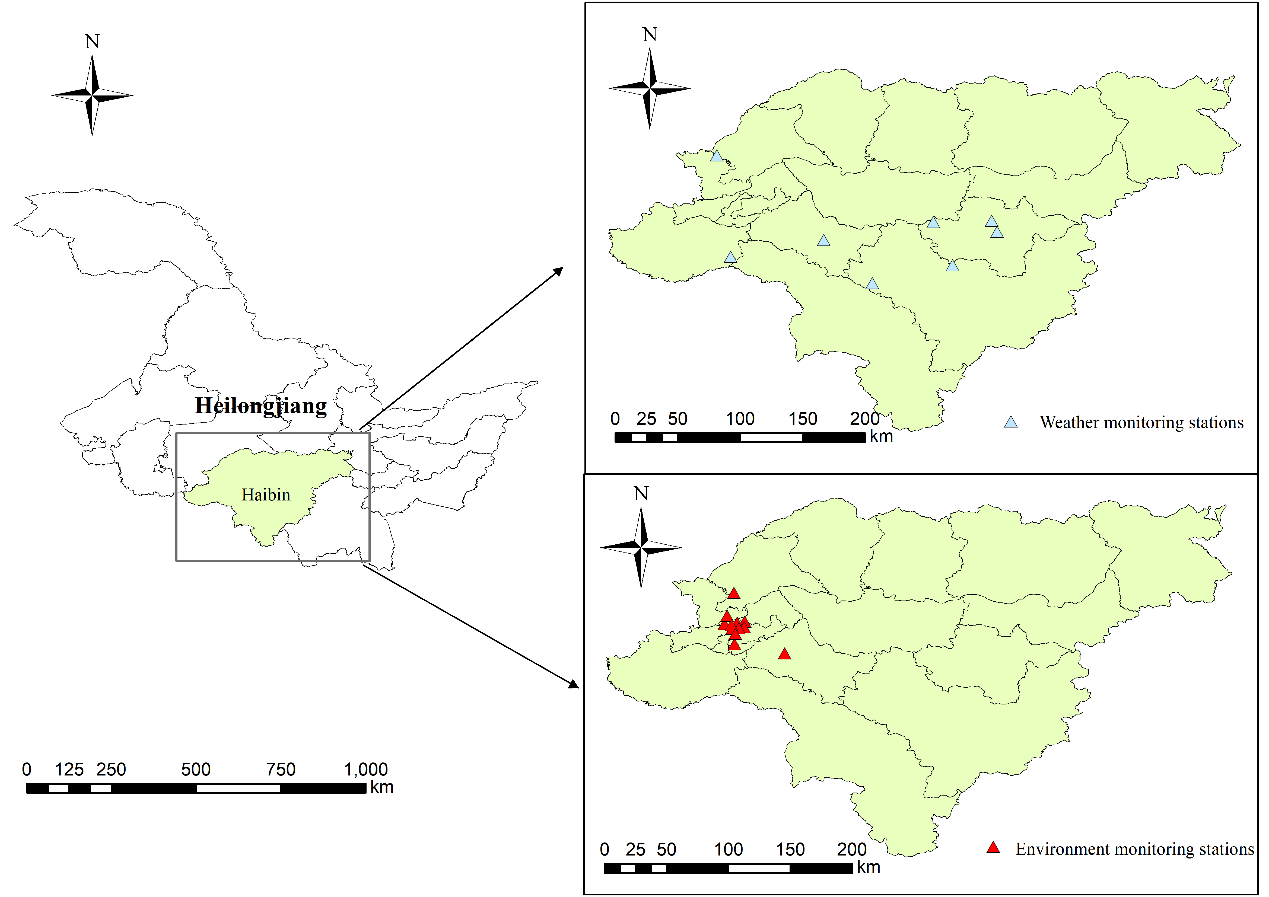


**Fig.S2.** Spatial distribution of air quality and meteorological monitoring stations in Harbin, Heilongjiang province (2013-2019)


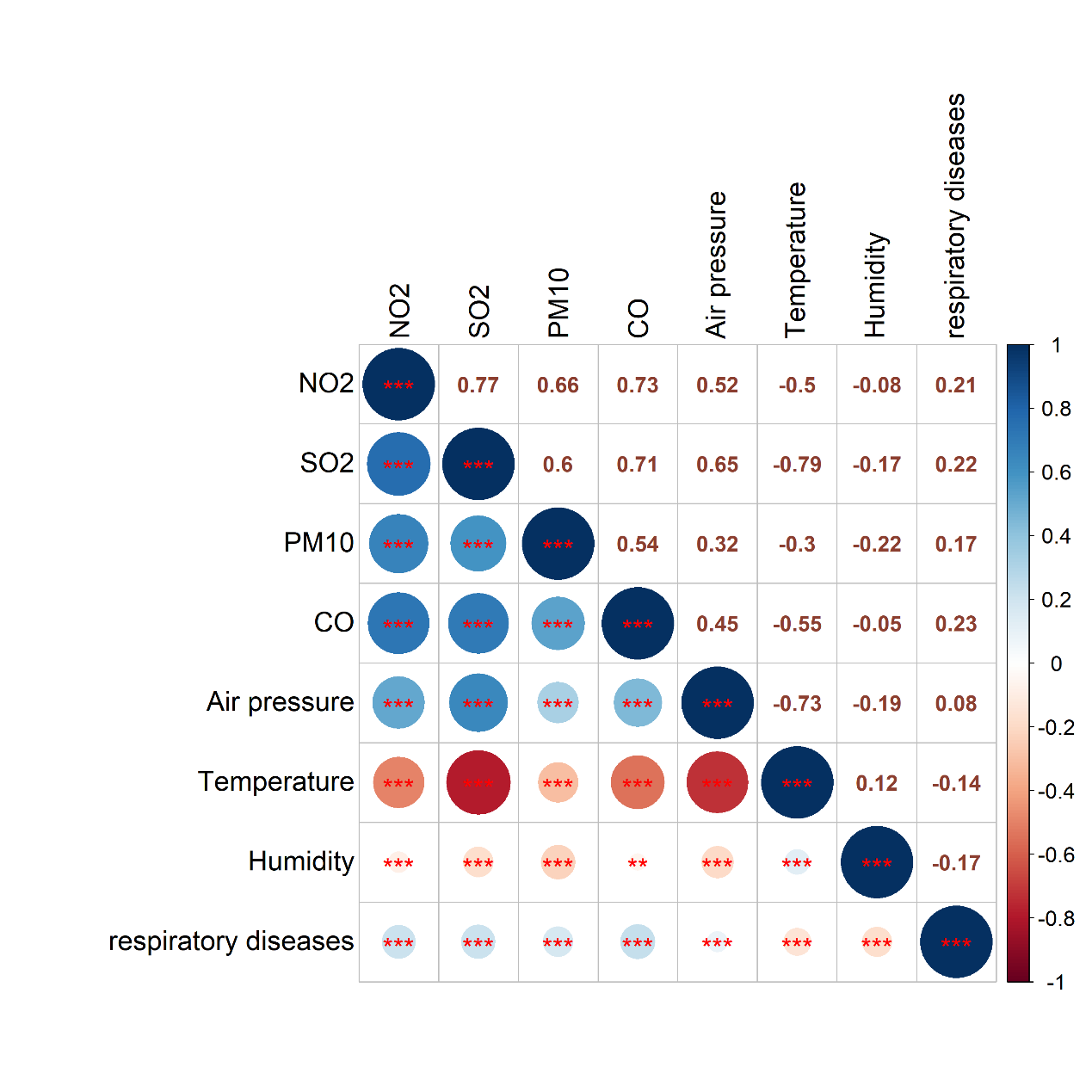


**Fig.S3.** Spearman rank correlation coefficients among air pollutants, meteorological data, and outpatient visits for respiratory diseases among children in Harbin, 2013-2019


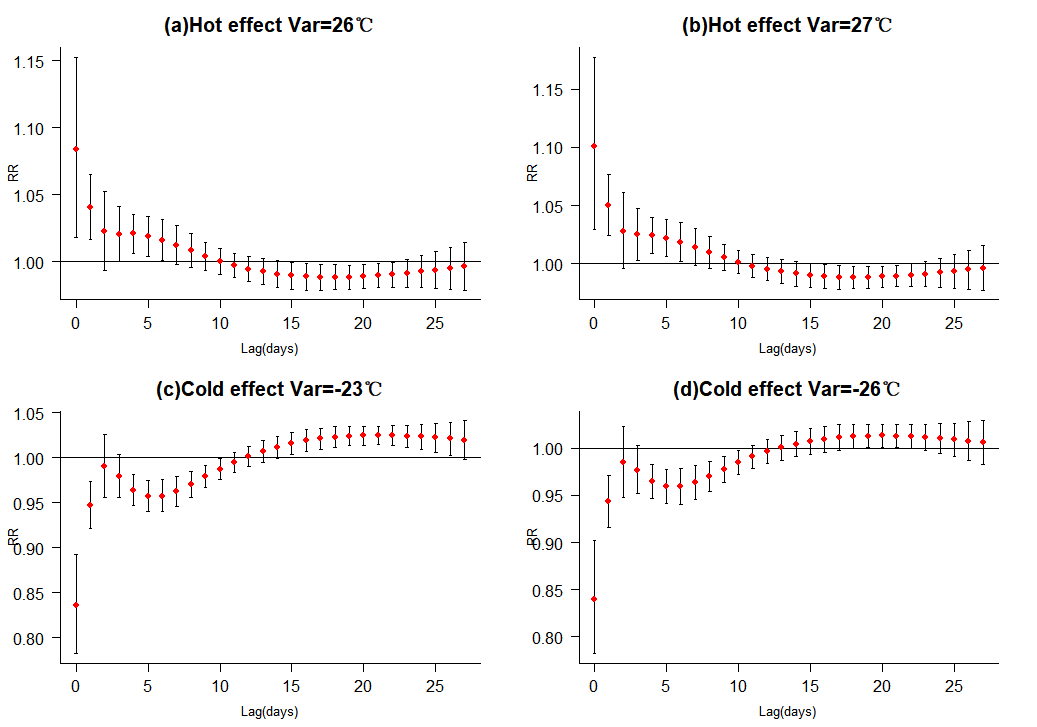


**Fig.S4.** (a) The RRs and 95% CIs of 97.5^th^ percentile for temperatures relative to median temperature (7 ℃) as a reference value on children’s outpatient visits for respiratory diseases at different lag days. (b) The RRs and 95% CIs of 99^th^ percentile for temperatures relative to median temperature (7 ℃) as a reference value on children’s outpatient visits for respiratory diseases at different lag days. (c) The RRs and 95% CIs of 2.5^th^ percentile for temperatures relative to median temperature (7 ℃) as a reference value on children’s outpatient visits for respiratory diseases at different lag days. (d) The RRs and 95% CIs of 1^st^ percentile for temperatures relative to median temperature (7 ℃) as a reference value on children’s outpatient visits for respiratory diseases at different lag days.

**
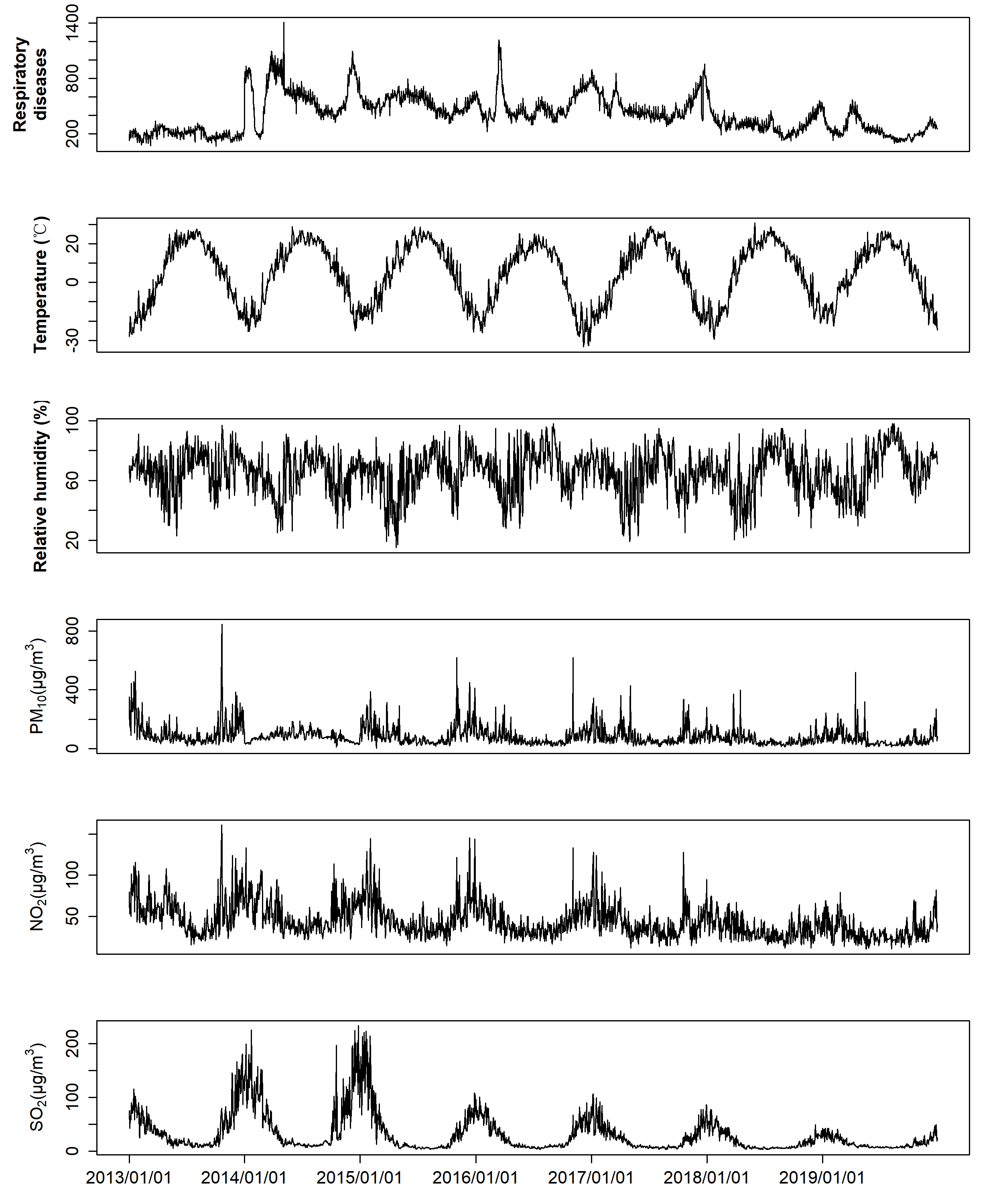
**

**Fig.S5.** Daily distribution of air pollutants, meteorological factors, and outpatient visits for respiratory diseases among children in Harbin, 2013-2019

**Table S1.** Variance inflation factor (VIF) values of independent variables included in the model in Harbin2013-2019

| Variables | VIF |
| --- | --- |
| NO_2_ | 3.44 |
| SO_2_ | 3.00 |
| PM_10_ | 1.89 |
| Temperature | 1.75 |
| Relative humidity | 1.02 |

**Table S2.** The cumulative effect (lag 0-10 days) of extreme temperatures relative to median temperature (7 ℃) as a reference value on children’s outpatient visits for respiratory diseases using different *df* for calendar time

| *df* | Temperature percentiles | RR (95% CI) |
| --- | --- | --- |
| 6 | 1^st^ | 0.59 (0.51, 0.68) |
|  | 2.5^th^ | 0.57 (0.50, 0.65) |
|  | 97.5^th^ | 1.32 (1.17, 1.50) |
|  | 99^th^ | 1.37 (1.20, 1.56) |
| 8 | 1^st^ | 0.67 (0.58, 0.78) |
|  | 2.5^th^ | 0.67 (0.59, 0.77) |
|  | 97.5^th^ | 1.45 (1.30, 1.63) |
|  | 99^th^ | 1.51 (1.34, 1.71) |
| 9 | 1^st^ | 0.75 (0.65, 0.86) |
|  | 2.5^th^ | 0.74 (0.65, 0.84) |
|  | 97.5^th^ | 1.47 (1.31, 1.64) |
|  | 99^th^ | 1.50 (1.33, 1.68) |
| 10 | 1^st^ | 0.67 (0.58, 0.77) |
|  | 2.5^th^ | 0.63 (0.56, 0.72) |
|  | 97.5^th^ | 1.25 (1.13, 1.39) |
|  | 99^th^ | 1.28 (1.14, 1.43) |

**Table S3.** The cumulative effect (lag 0-10 days) of extreme temperatures relative to median temperature (7 ℃) as a reference value on children’s outpatient visits for respiratory diseases using different *df* for relative humidity

| df | Temperature percentiles | RR (95% CI) |
| --- | --- | --- |
| 1 | 1^st^ | 0.61 (0.52, 0.71) |
|  | 2.5^th^ | 0.60 (0.52, 0.69) |
|  | 97.5^th^ | 1.39 (1.23, 1.56) |
|  | 99^th^ | 1.44 (1.27, 1.64) |
| 2 | 1^st^ | 0.59 (0.51, 0.69) |
|  | 2.5^th^ | 0.59 (0.51, 0.68) |
|  | 97.5^th^ | 1.40 (1.24, 1.58) |
|  | 99^th^ | 1.45 (1.28, 1.64) |
| 3 | 1^st^ | 0.59 (0.51, 0.69) |
|  | 2.5^th^ | 0.59 (0.51, 0.68) |
|  | 97.5^th^ | 1.40 (1.25, 1.58) |
|  | 99^th^ | 1.45 (1.28, 1.65) |
| 4 | 1^st^ | 0.59 (0.51, 0.69) |
|  | 2.5^th^ | 0.59 (0.51, 0.68) |
|  | 97.5^th^ | 1.40 (1.25, 1.58) |
|  | 99^th^ | 1.45 (1.28, 1.65) |
| 5 | 1^st^ | 0.59 (0.51, 0.69) |
|  | 2.5^th^ | 0.59 (0.51, 0.68) |
|  | 97.5^th^ | 1.40 (1.25, 1.58) |
|  | 99^th^ | 1.45 (1.28, 1.65) |
| 6 | 1^st^ | 0.60 (0.51, 0.70) |
|  | 2.5^th^ | 0.59 (0.52, 0.68) |
|  | 97.5^th^ | 1.40 (1.24, 1.57) |
|  | 99^th^ | 1.45 (1.27, 1.64) |
| 7 | 1^st^ | 0.60 (0.51, 0.70) |
|  | 2.5^th^ | 0.59 (0.52, 0.68) |
|  | 97.5^th^ | 1.40 (1.24, 1.58) |
|  | 99^th^ | 1.45 (1.28, 1.64) |

**Table S4.** The cumulative effect (lag 0-10 days) of extreme temperatures relative to median temperature (7 ℃) as a reference value on children’s outpatient visits for respiratory diseases using the covariate-adjusted standardization approach

| Model | Temperature percentiles | RR (95% CI) |
| --- | --- | --- |
| 1 | 1^st^ | 0.60 (0.51, 0.69) |
|  | 2.5^th^ | 0.59 (0.52, 0.68) |
|  | 97.5^th^ | 1.41 (1.26, 1.59) |
|  | 99^th^ | 1.47 (1.29, 1.67) |
| 2 | 1^st^ | 0.60 (0.52, 0.70) |
|  | 2.5^th^ | 0.60 (0.52, 0.69) |
|  | 97.5^th^ | 1.40 (1.24, 1.58) |
|  | 99^th^ | 1.45 (1.28, 1.65) |
| 3 | 1^st^ | 0.67 (0.57, 0.78) |
|  | 2.5^th^ | 0.67 (0.58, 0.77) |
|  | 97.5^th^ | 1.33 (1.20, 1.47) |
|  | 99^th^ | 1.37 (1.23, 1.52) |

*Note*: Model 1: Based on the main model (long-term trend, seasonal trend, day-of-week, holiday effect, PM_10_, SO_2_, and NO) further adjusted for CO; Model 2: Further adjusted for Air pressure and CO based on the main model. Model 3: Further adjusted for Air pressure, CO and PM_2.5_ based on the main model.
